# An Efficient Method for the Isolation and Cultivation of Hypothalamic Neural Stem/Progenitor Cells From Mouse Embryos

**DOI:** 10.3389/fnana.2022.711138

**Published:** 2022-02-04

**Authors:** Yichao Ou, Mengjie Che, Junjie Peng, Mingfeng Zhou, Guangsen Wu, Haodong Gong, Kai Li, Xingqin Wang, Peirong Niu, Songtao Qi, Zhanpeng Feng

**Affiliations:** ^1^Department of Neurosurgery, Nanfang Hospital, Southern Medical University, Guangzhou, China; ^2^The Laboratory for Precision Neurosurgery, Nanfang Hospital, Southern Medical University, Guangzhou, China; ^3^First Medical Institute, Southern Medical University, Guangzhou, China

**Keywords:** hypothalamus, mouse brain anatomy, neurodevelopment, neurogenesis, neural stem/progenitor cells

## Abstract

The hypothalamus is the key region that regulates the neuroendocrine system as well as instinct behaviors, and hypothalamic dysfunction causes refractory clinical problems. Recent studies have indicated that neural stem/progenitor cell (NSPC) in the hypothalamus play a crucial role in hypothalamic function. However, specific hypothalamic NSPC culture methods have not been established, especially not detailed or efficient surgical procedures. The present study presented a convenient, detailed and efficient method for the isolation and cultivation of hypothalamic NSPCs from embryonic day 12.5 mice. The procedure includes embryo acquisition, brain microdissection to quickly obtain hypothalamic tissue and hypothalamic NSPC culture. Hypothalamic NSPCs can be quickly harvested and grow well in both neurosphere and adherent cultures through this method. Additionally, we confirmed the cell origin and evaluated the proliferation and differentiation properties of cultured cells. In conclusion, we present a convenient and practical method for the isolation and cultivation of hypothalamic NSPCs that can be used in extensive hypothalamic studies.

## Introduction

The hypothalamus is one of the most important and complicated parts of the brain, and it regulates various essential biological processes, including energy homeostasis, maintenance of the internal environment, survival behavior, thermoregulation, aging, and circadian rhythms (Sternson, 2013; Saper and Lowell, 2014). Additionally, hypothalamic injuries may lead to refractory complications, especially central diabetes insipidus, obesity, growth retardation in children, and gonadal dysfunction (Honegger et al., 1999; Feng et al., 2018). In addition to exogenous supplementation for certain hormone deficiencies, an efficient method to improve hypothalamic dysfunction is still unclear. A few studies have indicated that complicated hypothalamic function can be precisely modulated by neurogenesis in the hypothalamus (Czupryn et al., 2011; Zhang et al., 2017). However, efforts are needed to determine how hypothalamic neural stem/progenitor cell (NSPCs) regulate hypothalamic function and how NSPCs communicate with surrounding cells in the hypothalamus. To understand the development of hypothalamus and the role of neurogenesis in hypothalamic function regulation, it is critical to culture and study hypothalamic NSPCs.

Cell cultivation is a widely used and effective method to study the long-term proliferation and multilineage differentiation properties of NSPCs *in vitro*. Isolation and culture methods for NSPCs have been established over four decades in an increasingly region-specific manner and have included various species and developmental stages ranging from chick embryos to adult mammals (Sensenbrenner et al., 1978; Gensburger et al., 1987; Reynolds and Weiss, 1992; Fischer et al., 2011). However, compared to other brain regions where neurogenesis occurs, only a few studies have focused on culturing NSPCs from the hypothalamus (Wataya et al., 2008; Cariboni et al., 2014; Kano et al., 2019; Nesan et al., 2020). However, in previous studies, the techniques to acquire hypothalamic NSPCs have been difficult to repeat due to difficult dissection methods, which may impair hypothalamic integrity or be limited by the availability of a cell sorting apparatus. Moreover, details about how to quickly obtain hypothalamic tissue were not mentioned in previous studies.

In this article, we introduced a convenient and reproducible method to quickly harvest and culture hypothalamic NSPCs from embryonic day 12.5 (E12.5) mouse embryos.

## Materials and Methods

### Animals and Ethics

C57BL/6 female mice that were pregnant with 12.5-day-old embryos were purchased from the Experimental Animal Center of Southern Medical University (Guangzhou, China) to generate hypothalamic NSPC cultures. Embryos in one dam were enough to establish primary hypothalamic stem/progenitor cell cultures. All experiments strictly followed the guidelines of the Ethics Committee of Nanfang Hospital of Southern Medical University.

### Hypothalamic Neural Stem/Progenitor Cell Culture Preparation

The root of a 30-gauge needle with a sterile 1 ml disposable plastic syringe was bent at an angle of approximately 45° using forceps and by clamping the middle of the needle. The tips of 1 ml pipette tips were cut off transversely before sterilization.

The neuronal cell isolation enzyme contained 2 ml of DMEM, 200 μl of 10 mg/ml DNase and 2 ml of 0.25% trypsin.

For adherent cultures, cell culture dishes were coated with poly-D-lysine (PDL, 20 μg/ml, Sigma-Aldrich) and incubated overnight at 4°C. The next day, the PDL was washed out with PBS solution three times. Culture dishes were coated again with laminin (10 μg/ml, Sigma-Aldrich) and stored at 4°C overnight. The dishes were washed three times to wash out the laminin before the cells were plated.

Media was prepared as follows: neural stem/progenitor cell media (NSPC media) contained neurobasal medium (Gibco) with 2% B27 supplement (50×) (Gibco), 20 ng/ml of EGF (Pepro Tech), 20 ng/ml of FGF (Pepro Tech) and 2 mM GlutaMAX (Gibco). Differentiation media contained neurobasal medium with 1 μM retinoic acid, 2% B27 supplement (50×), 2 mM GlutaMAX, and 0.5% fetal bovine serum (FBS).

### Harvesting Embryos

(1) The donor mouse was anesthetized with 1% sodium pentobarbital (50 mg/kg) through intraperitoneal injection. In this step, we ensured that the dam reached an adequate anesthetic state by observing the absence of a righting reflex, a slowed breathing rate and loss of the paw withdrawal reflex.
(2) The mouse was cervically dislocated.
(3) The dam was soaked in 75% ethanol for 1 min for disinfection.

*In the following steps, all the surgical instruments were sterilized, and a pair of sterile surgical gloves was required*.

(4) Forceps were used to move the dam onto sterile gauze on a predisinfected operating table.
(5) Forceps were used to hold the lower part of the abdominal skin, and then the abdominal cavity was transversely opened by scissors to expose the uterine horns. The uterus containing the string of embryos was separated from the abdomen by using another set of scissors to cut off all surrounding structures, including the mesometrium, blood vessels and cervix.
(6) The uterus was transferred into sterilized and chilled Hank’s Balanced Salt Solution (HBSS, Gibco) containing 2% penicillin–streptomycin solution (Gibco) in a new 10 cm Petri dish that was stored on an ice pack.

*The following steps were performed on a superclean bench, and another set of sterilized surgical instruments and a pair of new surgical gloves were needed*.

**FIGURE 1:**
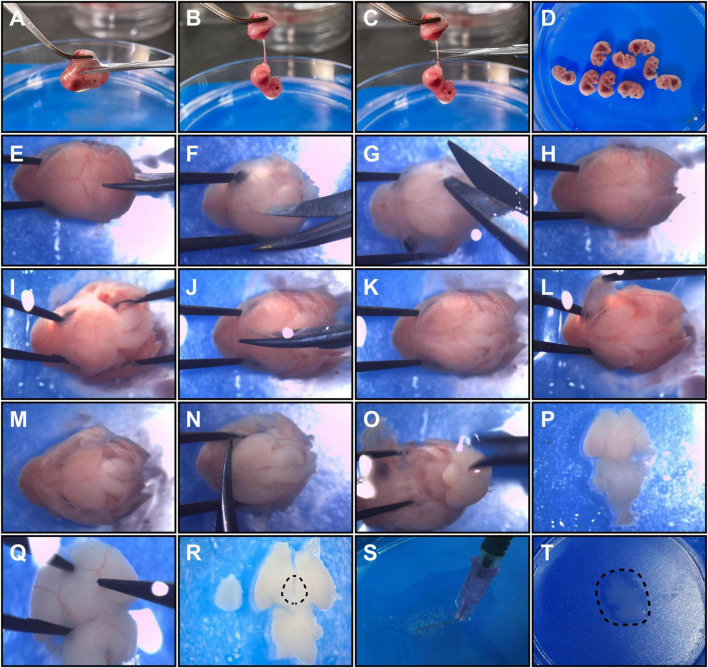
Detailed operation procedures to harvest hypothalamic tissue from E12.5 embryos. Panels **(A–D)** show embryo acquirement procedures. **(A)** The placenta was clamped, and a cut was made to open the fetal membrane. **(B)** An exposed embryo linked to the umbilical cord is shown. **(C)** The umbilical cord was cut close to the embryo. **(D)** The embryos were collected in a new dish. Panels **(E–P)** show the brain microdissection procedures. **(E,F)** Head fixation and sagittal cutting of occipital and parietal bones are shown. **(G)** A coronary cut was performed along the lambdoid suture. **(H)** The separated occipital and parietal bones are shown. **(I)** The occipital bones were detached from the brain. **(J)** The frontal bones were sagittally cut. **(K)** A head is shown with the cranial bones incised. **(L)** The parietal bones and the frontal bones were detached from the brain. **(M)** The exposed dorsal side of the brain is shown. **(N)** The olfactory bulb was cut to free the brain. **(O)** The fetal brain was isolated from the skull. **(P)** A ventral view of the fetal brain is shown. **(Q)** Removement of hypothalamic surrounding membranes and vessels. **(R)** The dotted line shows the relative dissection margins for hypothalamus microdissection. **(S)** The brain tissue was minced into small pieces with a bent needle. **(T)** The final hypothalamic tissue fragments obtained are shown.

(7) Scissors were used to separate embryos one by one through amniotic sac interspaces.
(8) The placenta of each embryo was lifted with micro ophthalmic forceps, and an opening was cut in each fetal membrane (Figure 1A). One blade of the scissors was inserted into the opening and moved outward slightly to expose the fetus to the outside without difficulty (Figures 1A,B). The umbilical cord was cut (Figure 1C), and the embryo was transferred to another dish with chilled DMEM (Gibco) on an ice pack (Figure 1D). The same steps were repeated to collect all remaining embryos.

(9) The fetal heads were cut out one by one and transferred to a new Petri dish with chilled HBSS/1% penicillin–streptomycin.

### Microdissection of Fetal Mouse Brains

*The following operations were performed under a stereo light microscope. A blue ice pack is recommended to keep the Petri dish cold for the high-contrast operation*.

**FIGURE 2:**
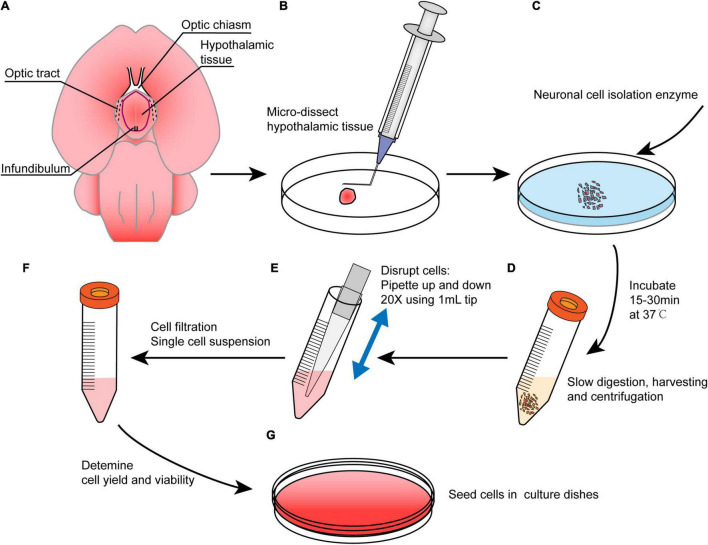
An overview of the main procedures in this method. **(A)** Brain preparation in the ventral side-up position to show hypothalamic borders. Red line shows the dissection margins. **(B)** Brain microdissection was performed to obtain hypothalamus tissue, and the tissue was minced with a bent needle. **(C)** The tissue was digested with neuronal isolation enzyme at 37°C for 15–30 min. **(D)** The digestion reaction was slowed with DMEM followed by centrifugation, and the supernatant was discarded. **(E)** The pellet was resuspended in NSPC medium and triturated. **(F)** The cells were filtered to obtain a single cell suspension. **(G)** Cells were cultured in NSPC medium at a density of 1–3 × 10^5^ cells/ml after a viability assay.

(1) The head of each fetus was held by clamping the eye sockets (or the nasal part) with forceps, and a blade of microscissors was inserted into the gap between the occipital bone and medulla (Figure 1E).
(2) The occipital bone and parietal bones were sagittally incised (Figure 1F). The microscissors were not inserted too deep to prevent brain damage.
(3) The occipital bone and parietal bones were isolated by coronary incision along the lambdoid suture (Figure 1G).
(4) Each head was held and the occipital bone and its accessory structures were carefully detached from the brain tissue by microforceps (Figures 1H,I).
(5) Separation of the head bones was continued by sagittal incision reaching the nasal point by microscissors (Figure 1J).
(6) The parietal bones and the frontal bones were detached by microforceps to totally expose the dorsal side of each fetal brain (Figures 1K–M).
(7) Microforceps were inserted beside the olfactory bulb to immobilize the head, and microscissors were used to dissect the olfactory bulb axially close to the telencephalon to free the brain from the head (Figure 1N).
(8) Opened microforceps were inserted superficially into the spaces between the basal surfaces of the telencephalon and the anterior skull base and were then gently moved backward to isolate the brain (Figure 1O). To keep the hypothalamus intact, we were careful not to insert the microforceps too deeply or to pull them out too quickly. After this step, the fetal brain was in the ventral side-up position (Figure 1P).
(9) The membrane structures and vessels surrounding the hypothalamus were ready to uncover (Figure 1Q). Specifically, the membranes at the border of the hypothalamic area were clamped by inserting the tip of the microforceps into cisterns around the hypothalamus (there are some regions in which membranes are easy to clamp, e.g., the internal carotid artery cistern and the interpeduncular cistern). Then, the membranes were gently picked up with microforceps, and the vessels were naturally separated along the membranes.
(10) The hypothalamus was dissected with microforceps along the following approximate margins: anterior to the chiasm, posterior to the posterior aspect of the infundibulum, and lateral to the optic tract (Figures 1R, 2A). And the depth for dissection was along the horizontal plane of brain, close to optic chiasm and optic tract. The optic chiasm and optic tract were not included in the dissection process.

### Preparation of Single Cell Suspensions

(1) The dissected hypothalamic tissues were collected into a new ice-cold 10 cm Petri dish (Figures 2A,B).
(2) The tissue was minced using a bent needle (Figures 1S, 2B) as described in a previous study (Kim et al., 2016). In the end, the tissue was cut into small pieces less than 0.5 mm^3^ (Figure 1T).
(3) Neuronal cell isolation enzyme was added, and the tissue was incubated at 37°C for 15–30 min (Figure 2C). The dish was shaken every 5 min. Then, 1 ml of DMEM/10% FBS was added to slow the enzyme activity (Figure 2D).
(4) The tissue was gently pipetted up and down 10–20 times using a 1 ml cut pipette tip, and the fluid was collected into a 15 ml centrifuge tube (Figure 2E). The remnant tissue at the bottom of the Petri dish can be picked up by rinsing with DMEM/10% FBS again if it is too much.
(5) The cell suspension was centrifuged at 1000 rpm for 5 min. The supernatant was removed and then 4–5 ml of chilled DMEM was added to resuspend the cells by pipetting 20–30 times using a 1 ml cut pipette tip. The pipetting procedure was performed slowly to avoid the introduction of gas bubbles.
(6) The cell suspension was filtered using a 70 μm sterile syringe filter into a 50 ml centrifuge tube to filter out large tissue fragments, and the filtrates were transferred into a new 15 ml centrifuge tube.
(7) The filtrates were centrifuged at 1000 rpm for 3–5 min. The supernatant was discarded and 2 ml of 37°C prewarmed NSPC media was added to generate a single cell suspension (Figure 2F).
(8) Live cells were counted with a hemocytometer and 0.4% trypan blue solution.
(9) Low attachment dish and coated dish were used for neurospheres and adherent cultures, respectively, to plate the cells at a density of 1–3 × 10^5^ cells/ml (Figure 2G).
(10) The cells were cultured at 37°C in a 5% CO_2_ incubator.

### Cell Culture Maintenance and Passaging

*In the following steps, the culture media and PBS were prewarmed in a 37°C water bath unless otherwise stated*.

To culture neurospheres, 50% of the NSPC media volume was refreshed in the first 48 h, and the media was refreshed the same way every 2–3 days thereafter. Neurospheres formed after approximately 5–10 days. To passage neurospheres, the neurospheres were triturated by pipetting up and down 30–40 times for dissociation until no cell clusters were visible and then were transferred to a 15 ml centrifuge tube. The suspension was centrifuged at 1000 rpm for 3 min. After discarding the supernatant, 1 ml of NSPC medium was added to resuspend. The total cells number was counted, and the cells were plated at 1–3 × 10^5^ cells/ml as secondary neurospheres. The same procedure was performed for neurospheres that were generated later.

For adherent cultures, 50% of the culture medium volume of primary hypothalamic NSPCs was refreshed every 2–3 days. When the cultures reached 70–80% confluence, they were passaged. For passaging, the NSPC medium was discarded from the coated dish, and the dish was washed three times with PBS. Accutase (Gibco) was added to cover the adherent NSPCs surface, and the cells were incubated for 3–5 min in a standard incubator. Two milliliters of DMEM was added to slow the enzyme digestion and was gently pipetted up and down for cell detachment. Then, the detached cells were transferred to a 15 ml centrifuge tube. The centrifugation and plating processes were the same as those described above for the neurospheres except for the dish type.

### Hypothalamic Neural Stem/Progenitor Cell Proliferation and Differentiation

Preparation: an adequate number of confocal dishes were coated with PDL/laminin for subsequent use.

A proliferation assay was performed on the neurospheres and adherent cells. For adherent cell culture, NSPCs were plated in coated confocal dishes at 1 × 10^5^ cells/ml and incubated for 24 h. For neurospheres, the neurospheres were plated in coated confocal dishes. BrdU (Sigma-Aldrich, final concentration: 5 μM) was added to the confocal dishes for both culture types followed by incubation for 6 h at 37°C before fixation.

To test differentiation properties, NSPCs were plated in coated confocal dishes with NSPC media at a density of 3–6 × 10^4^ cells/ml and incubated at 37°C. Then, 50% of the NSPC medium was replaced with differentiation medium on the second day. During the following media change, 100% of the medium was replaced with differentiation medium every 2–3 days after washing the dishes three times with PBS. The whole differentiation time was 5–7 days. Finally, the cells were fixed for immunostaining.

### Quantitative Real-Time PCR

Total RNA was extracted from E12.5 mice brain tissue and cultured NSPCs of passage 3–5, using TRIzol reagent (Takara). And cDNA was synthesized using Evo M-MLV RT Kit (Accurate Biology, China). For quantitative real-time PCR (qRT-PCR), cDNA was amplified using SYBR ^®^ Green Premix Pro Taq HS qPCR Kit II (Accurate Biology, China). The relative expression level of mRNA was normalized to Gapdh. The specific primers used for Gapdh, Nkx2.1, Six3, Ascl1, and Pax6 are shown in Supplementary Table 1.

### Immunofluorescence

For BrdU immunostaining, the cells were incubated at 37°C in 2 N HCl for 15 min and in 0.1 M borate buffer (pH 8.5) for 10 min. For immunofluorescence staining, the cells were rinsed three times with PBS for 5 min each. PBS was discarded, and the cells were blocked with 5% goat serum containing 0.5% Triton X-100 at 37°C for 60 min. The cells were incubated with primary antibodies at 4°C overnight. The cells were washed three times in PBST (0.2% Triton X-100 in 1× PBS) for 5 min each with slow shaking. The cells were incubated with secondary antibodies in darkness at 37°C for 1 h. The cells were washed in PBST six times for 10 min each and mounted by the dropwise addition of fluorescence mounting medium containing DAPI (Abcam) to the cell surface for imaging. Immunostained cells were viewed and imaged with a Zeiss confocal microscope (Zeiss LSM 980) and counted manually by using ImageJ software (NIH). The primary antibodies used were as follows: mouse anti-Nestin (CST), rabbit anti-Sox2 (CST), rabbit anti-NKX2.1 (CST), rabbit anti-SIX3 (Novus), rabbit anti-ASCL1 (Abcam), rabbit anti-PAX6 (Abcam), rabbit anti-Ki67 (CST), mouse anti-BrdU (Roche), rabbit anti-Tuj1 (Abcam), rabbit anti-Map2 (CST), rabbit anti-GFAP (Abcam), and mouse anti-Olig2 (Millipore).

### Statistical Analysis

SPSS and GraphPad Prism software were used for statistical analysis. Data are presented as the mean ± SEM. Data were submitted to Kolmogorov–Smirnov and Shapiro–Wilk normality test to identify the distribution. Two-tailed Student’s *t*-test and Mann–Whitney *U* test were used for comparison between two groups. Differences were considered statistically significant at *P*-values < 0.05.

## Results

A novel method was used to acquire and culture enough living embryonic hypothalamic NSPCs for further hypothalamic study. The whole process included pregnant mouse surgery to obtain mouse embryos, fetal brain acquisition, microdissection of hypothalamus tissue and cell culture (Figure 2). The surgical instruments used in this article were standard microsurgical instruments, and no other special equipment was needed. To preserve cell viability as much as possible, refined operation skills were performed, as operation time was reduced and tissue impairment was minimized (Figure 1). In our method, only two cuts were needed to isolate one embryo (Figures 1A–C). To preserve the intact hypothalamic tuberal area, microdissection was performed anterior to the chiasm, posterior to the posterior aspect of the infundibulum, and lateral to the optic tract. The dissection margins were outlined ventrally (Figures 1R, 2A), which was slightly smaller than the real hypothalamus border. A bent needle was used to mince hypothalamic tissue into small pieces (Figures 1S,T). The membranes and vessels covering the base of the brain were completely removed (Figures 1Q,R). The whole operation time from embryo acquisition to cell seeding was 1.5–2 h. After single cell suspension prepared, the percentage of viable cells was considerable (93.76 ± 0.93%, *n* = 7) assessed by trypan blue staining.

**FIGURE 3:**
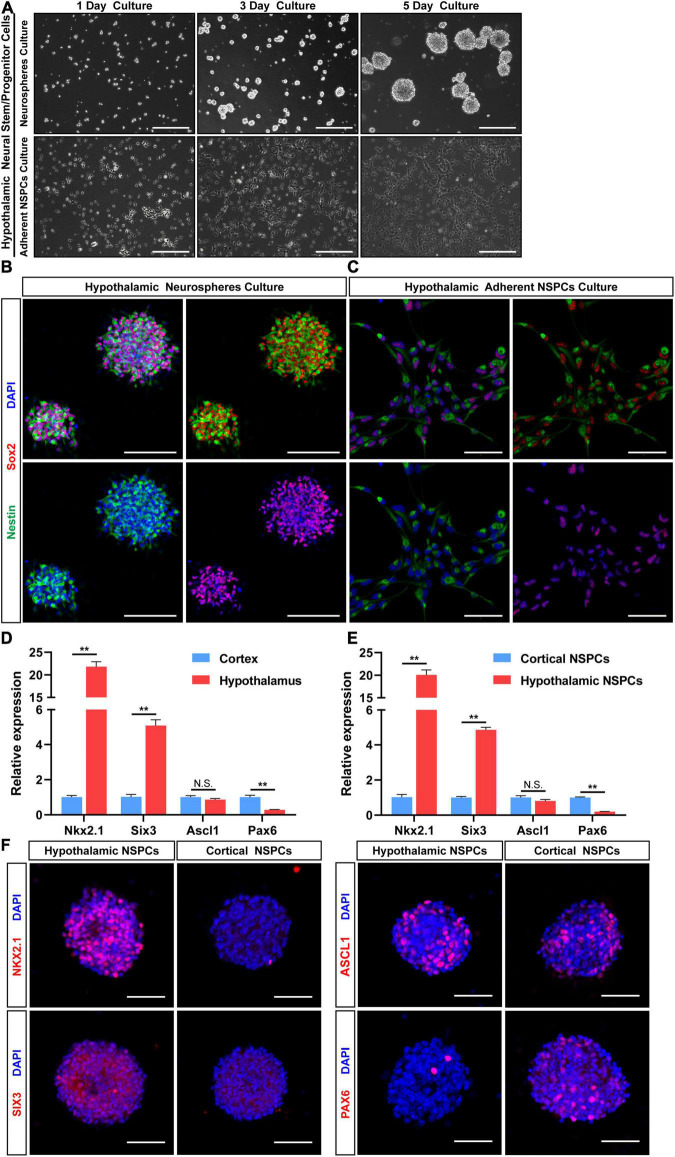
Maintenance of hypothalamic NSPCs as neurospheres and adherent NSPC cultures. **(A)** Images of hypothalamic primary NSPCs cultured as neurospheres and monolayer cells for 1, 3, and 5 days. Scale bar: 200 μm. **(B,C)** Double immunostaining for Sox2 (red) and Nestin (green) of hypothalamic primary NSPCs in two culture methods. Scale bar **(B)** 100 μm, scale bar **(C)** 50 μm. **(D)** qRT-PCR analysis of the E12.5 hypothalamic and cortical tissue expression levels of Nkx2.1, Six3, Ascl1, and Pax6. **(E)** qRT-PCR analysis of the cultured hypothalamic and cortical NSPCs (passage 3–5) expression levels of Nkx2.1, Six3, Ascl1, and Pax6 (*n* = 3 in each group). Data are showed as mean ± SEM. **P < 0.01 by two-tailed Student’s *t*-test. **(F)** Immunostaining for NKX2.1, SIX3, ASCL1, and PAX6 of hypothalamic and cortical neurospheres (passage 3–6). Scale bar: 50 μm.

**FIGURE 4:**
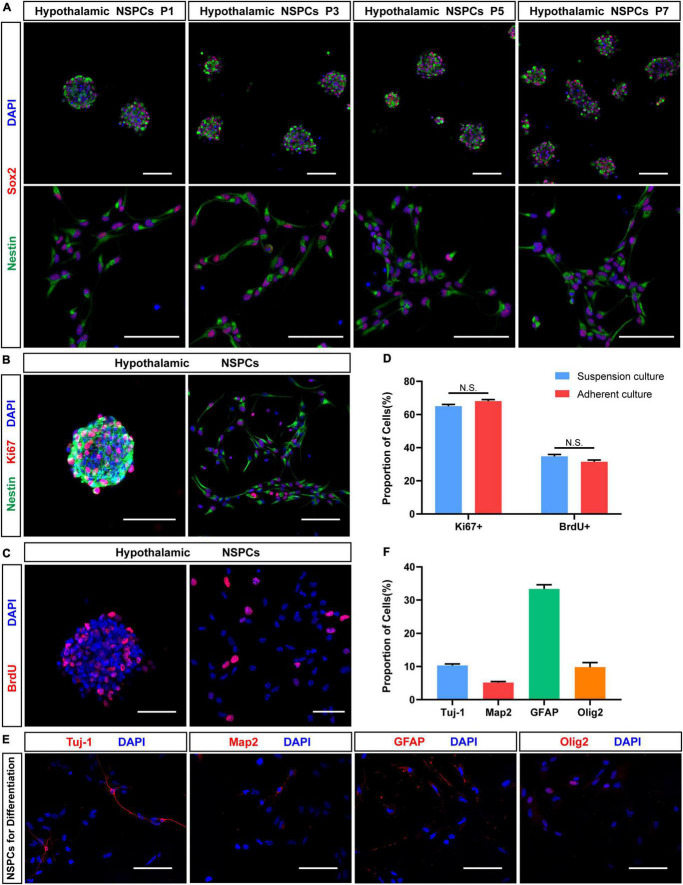
Proliferation and neuronal differentiation assay in hypothalamic NSPCs. **(A)** Immunostaining for Sox2 (red) and Nestin (green) of hypothalamic NSPCs in different passage (P1, P3, P5, P7). Scale bar: 75 μm. **(B)** Immunostaining for Nestin (green) and Ki67 (red) of P3–5 hypothalamic NSPCs. Scale bar: 100 μm. **(C)** Immunostaining for BrdU (red) after 6 h of BrdU incorporation of P3–5 hypothalamic NSPCs. Scale bar: 50 μm. **(D)** The percentage of Ki67 and BrdU immune-positive cells in **(B,C)** (*n* = 6 in each group; not significant by two-tailed Student’s *t*-test). **(E)** Immunostaining for Tuj-1, Map2, GFAP, and Olig2 of P3–6 hypothalamic NSPCs after 5–7 days differentiation. Scale bar: 100 μm. **(F)** The relative percentage of immunopositive cells in **(E)** (*n* = 6 in each group).

When using NSPC culture medium, the cells isolated from the embryonic hypothalamus generated both neurospheres and adherent cells using the different culture methods in this article (Figure 3A). Both neurospheres and adherent cells coexpressed the classical neural stem cell markers Sox2 and Nestin (Figures 3B,C). The formation of neurospheres and the expression of stem cell markers suggested that the NSPCs were successfully cultured using our method. Next, we performed qRT-PCR and immunostaining to verify whether cultured NSPCs were derived from the hypothalamus. The Nkx2.1 and Six3 gene are involved in ventral hypothalamus patterning at early stage (Markakis, 2002; Shimogori et al., 2010; Kim et al., 2020). And Ascl1 is a critical gene for hypothalamic neuroendocrine cells formation (Toda et al., 2017). Q-PCR result showed the high levels of Nkx2.1 and Six3 mRNA expression and low levels of Pax6 mRNA expression in E12.5 hypothalamic tissue compared with E12.5 cortical tissue (Figure 3D). The expression of Ascl1 gene was not significantly different between two groups (Figure 3D). The same gene expression trend of tissue was found in cultured NSPCs of passage 3–5, by qRT-PCR (Figure 3E) and immunostaining (Figure 3F). These results indicate that hypothalamic NSPCs cultured by our method had good regional specificity. Moreover, the stemness property was maintained after serial passages because the cell morphology almost unchanged and stem/progenitor cell markers Nestin and Sox2 were expressed (Figure 4A), suggesting that these cells can be regarded as a stable source to study the hypothalamus.

To test the proliferation properties of hypothalamic NSPCs, immunostaining of cell proliferation marker Ki67 and BrdU (5-bromo-2′-deoxyuridine) was performed (Figures 4B,C). Most of hypothalamic NSPCs express Ki67 (suspension culture: 65.03 ± 1.06%, *n* = 6; adherent culture: 68.11 ± 0.90%, *n* = 6; Figure 4D). After 6 h of BrdU labeling, the hypothalamic NSPCs generated by the two culture methods showed a high BrdU-positive percentage (suspension culture: 34.77 ± 1.13%, *n* = 6; adherent culture: 30.34 ± 1.58%, *n* = 6; Figures 4C,D). In addition, Ki67 or BrdU positive percentages were not significantly different between the two culture methods (Figure 4D), indicating that both neurospheres and adherent NSPCs are good forms to maintain proliferating hypothalamic NSPCs. To test the differentiation potential of hypothalamic NSPCs, the differentiation experiments were applied for 5–7 days. Immunostaining images (Figures 4E,F) showed that the cultured cells had the ability to differentiate into Tuj-1+ immature neurons (10.33 ± 0.45%, *n* = 6) and MAP2+ mature neurons (5.14 ± 0.79%, *n* = 6). Apart from neuron fate, we also found GFAP+ astrocytes (33.34 ± 1.28%, *n* = 6) and Olig2+ oligodendrocytes (9.81 ± 1.38%, *n* = 6) after differentiation (Figures 4E,F). Altogether, the hypothalamic NSPCs exhibit multilineage differentiation potential. These results suggest that by using our methods, hypothalamic NSPCs can be successfully isolated and maintained and that these cells have the potential to generate multiple types of cells of nervous system, especially neurons, which can be used in further *in vitro* and *in vivo* studies.

## Discussion

Here, we applied a practical method to successfully culture embryonic hypothalamic NSPCs. Our method is mainly based on knowledge of hypothalamus development and relative anatomy.

Neurogenesis first occurs in the hypothalamus, followed by NSPCs migration from the matrix layer surrounding the third ventricle. Once migration begins, the cells arrive at the nucleus in 24 h (Shimada and Nakamura, 1973). Although different regions have different initial neurogenesis times and durations, most of the NSPCs aggregate into the hypothalamic nucleus during E10–E14, and the early peak neurogenesis time (NSPCs proliferation phase) of the hypothalamus is E12.5 (Shimada and Nakamura, 1973; Markakis, 2002; Shimogori et al., 2010). Hence, to acquire more hypothalamic NSPCs and ensure that most of the cells acquired were at the proliferating and undifferentiated stage, E12.5 mouse embryos were used to perform our experiment instead of other timepoints.

A detailed ventral view of the rat hypothalamus containing intact membrane structures and cranial nerves was presented in our previous study (Ou et al., 2020). However, the embryo brain structure is different from the adult brain structure. At different stages, the embryonic brain has a different morphology. Although a ventral approach to extract the mouse embryo hypothalamus had been performed in a previous report (Cariboni et al., 2014), the detailed operation procedure and the precise extraction hypothalamic border were not mentioned. Moreover, the E14.5 mouse embryos used in previous studies failed to match the peak time of hypothalamic NSPCs proliferation. We therefore established a standard operating procedure to minimize variations between the primary cultures generated by different operators.

A full understanding of embryonic brain anatomy, especially in embryonic mice, is the basis for the precise and rapid isolation procedures described herein. An embryonic mouse histology atlas (Schambra, 2008) was reviewed before performing microsurgery to recognize the relative hypothalamic borders. In a ventral view of an E12.5 mouse brain, the rostral boundary of the hypothalamus is the laminal terminals, which is a thin plate near the medial ganglionic eminence (MGE). Through the median of the laminal terminals, the optic recess can be clearly observed, which can serve as a symbol to separate the hypothalamus and the MGE. Notably, in the embryonic stage, the MGE is one of the main stem cell pools to generate cortical inhibitory neurons (Mayer et al., 2018). Thus, to prevent tissue contamination by the MGE, a microdissection procedure should be performed carefully to not extend beyond the laminal terminals. The lateral boundaries of the hypothalamus consist of the lateral border of the anterior hypothalamic area rostrally, which is surrounded by the optic tract and ventromedial hypothalamic nucleus caudally. Caudally, the posterior edge of the mammillary bodies separates the hypothalamus and midbrain. And on the dorsal side, the hypothalamus is adjacent to the prethalamus. Interestingly, the Willis’ circle surrounds the ventral outline of the hypothalamus. Hence, two approximate surgical boundaries were chosen to isolate the hypothalamus from the ventral surface of the brain. To isolate the entire hypothalamus with all its nuclei, the surgical incision boundary was near Willis’ circle. However, inevitably, other nearby brain tissues will be included, which will decrease the specificity of the tissue and may cause unexpected influences on further tissue culture studies. For these reasons, a smaller boundary was applied in this article. We picked the hypothalamus region that was anterior to the chiasm, posterior to the posterior aspect of the pituitary stalk, and lateral to the optic tract.

While functionally, the hypothalamus is divided into four regions (preoptic, supraoptic, tuberal and mammillary), their cell derivations are a little different. When hypothalamic neurons are generally considered to derive from proliferative zone of third ventricle, preoptic area receives neurons both from proliferative zone of third ventricle and lateral ventricles (Paredes, 2003). At stage E12.5, axons and their growth cones derived from retinal ganglion cells first arrive at the diencephalon and begin to cross the midline to construct the optic chiasm (Kruger et al., 1998). At this stage, the optic chiasm can be seen ventrally by microscopy and is the same as the initial portion of the optic tract, but fainter. The optic chiasm gives operators a natural anatomical delimitation to distinguish preoptic area and other hypothalamic regions. Since chiasm was seen as a dissection border, preoptic area was not included in tissue collection here. In this method, the intact mediobasal hypothalamus was acquired, and no region outside the hypothalamus was accepted. We hope that this method could help studies about the development of mediobasal hypothalamus.

Based on a previous fetal meninges study (Dasgupta and Jeong, 2019), the endothelial cells of blood vessels were derived from the mesoderm, while the three layers of meninges in the diencephalon were of neural crest origin. However, compared with the hypothalamus, the primitive meninges have different differentiation directions. Vessels and neural stem cells have tight mutual interactions that can affect the self-renewal, differentiation, and migration progress of neural stem cells (Segarra et al., 2019). Hence, we emphasized the importance of removing the membranes covering the base of the brain, especially the hypothalamus, to prevent culture contamination or cell growth interference by membrane or vessel cells.

During brain tissue microdissection, many protocols were inclined to use scissors or scalpel blades to cut brain tissue into pieces (Babu et al., 2011; Seibenhener and Wooten, 2012; Roqué and Costa, 2017). Only a few articles reported other methods for mincing, for instance, using a bent needle (Kim et al., 2016). However, unlike bulk tissue, the extracted tissue of each embryonic brain in this article was small. The average volume of these tissue was less than 2 mm^3^ (Figure 1R). As large dissection tools such as scissors was not flexible for this use, we choose a bent needle for microdissection. In our practice, a bent needle was more appropriate for mincing E12.5 hypothalamic tissue for its faster cutting speed and consistent final debris diameter. Moreover, the cell viability can be well maintained (93.76 ± 0.93% living cells assessed by trypan blue) after mincing brain tissue with a needle, and the operator would not feel uncomfortable.

Hypothalamic NSPCs from embryos can give rise to neurons, astrocytes, and oligodendroglia, which is similar to NSPCs in other brain regions. And hypothalamic NSPCs can also produce neuroendocrine cells *in vivo* and *in vitro* (Cariboni et al., 2014). These findings suggest that neurogenesis in the hypothalamus has different features from neurogenesis in other regions. In our study, we found that embryonic hypothalamic primary neurospheres could be easily generated within 10 days (commonly 5–7 days), which requires less time than adult NSPC culture (10 days to 3–4 weeks) (Robins et al., 2013; Zilkha-Falb et al., 2020). Consistent with previous studies (Robins et al., 2013; Cariboni et al., 2014), we found that hypothalamic NSPCs could be stably passaged more than 10 times. Nonetheless, we recommend using early generation of NSPCs for long term functional studies. All together, these properties indicate that our NSPCs may serve as available substitutes for previous studies.

This method can also be extended to the cultivation of adult hypothalamic NSPCs. The processes of brain isolation and the medium for cell proliferation and differentiation are applicable. However, due to the different characteristics between adults and embryos, some tips need to be noted when this method applied to adults. Noticeably, adult hypothalamic neurogenesis is maintained at low level under physiological conditions (Yoo and Blackshaw, 2018). For enriching more NSPCs at one time, several mice are needed. In addition, adult brain cells are vulnerable to ischemia or hypoxia. The time to dissect a single brain needs to be restricted after the neck is severed. Moreover, adult hypothalamic neurogenesis is mainly concentrated in the mediobasal hypothalamus (Yoo and Blackshaw, 2018). So, we recommend dissecting only the tissues around the third ventricle. For the dealing with highly developed blood vessels in adult brain, tearing off the membrane and vessels totally is required. And a trypsin with EDTA is recommended and the concentration of trypsin can be increased to quickly obtain single cell suspension. Furthermore, compared to embryonic culture, the generation time for primary adult neurosphere will be prolonged.

The harvest of hypothalamic NSPCs by our method is a useful tool to study hypothalamic functions and diseases. *In vitro* NSPC studies, such as coculture methods and culture of hypothalamic NSPCs with cytokines, are essential to understand the development of the hypothalamus and cell-to-cell connections in the hypothalamus. Moreover, neural stem/progenitor-cell transplantation in the hypothalamus is an underlying approach to investigate the mechanisms of hypothalamus-related phenomena, such as aging (Zhang et al., 2017) and obesity (Czupryn et al., 2011). In oncology studies, not only could they serve as a group of controls to study glioblastoma tumorigenicity (Liu et al., 2020), hypothalamic NSPCs could also be used to study the origin and mechanism of hypothalamic/optic pathway gliomas (Lee et al., 2012). However, our method also has limitations. We did not validate the differentiation potential to generate various hypothalamic neuroendocrine neurons, which was validated in other articles (Markakis et al., 2004; Wang et al., 2015; Kano et al., 2019) due to the long differentiation time (at least 3 weeks).

## Conclusion

In conclusion, we provided a convenient and practical method to obtain hypothalamic NSPCs with a detailed and efficient surgical procedure. This method may allow more researchers to obtain hypothalamic NSPCs for further related researches.

## Data Availability Statement

The raw data supporting the conclusions of this article will be made available by the authors, without undue reservation.

## Ethics Statement

The animal study was reviewed and approved by the Ethics Committee of Nanfang Hospital, Southern Medical University.

## Author Contributions

YO and MZ contributed to the conception and design of the study. YO, MC, and JP designed the figures, performed the experiments, and wrote the manuscript with supervision from XW, SQ, and ZF. HG, GW, KL, and PN did the investigation, and collected and analyzed the data. SQ, ZF, YO, JP, MZ, and XW contributed to the funding acquisition. All authors contributed to the article and approved the submitted version.

## Conflict of Interest

The authors declare that the research was conducted in the absence of any commercial or financial relationships that could be construed as a potential conflict of interest.

## Publisher’s Note

All claims expressed in this article are solely those of the authors and do not necessarily represent those of their affiliated organizations, or those of the publisher, the editors and the reviewers. Any product that may be evaluated in this article, or claim that may be made by its manufacturer, is not guaranteed or endorsed by the publisher.
